# Work-related and personal factors in shoulder disorders among electronics workers: findings from an electronics enterprise in Taiwan

**DOI:** 10.1186/s12889-021-11572-4

**Published:** 2021-08-09

**Authors:** Po-Ching Chu, Tyng-Guey Wang, Yue Leon Guo

**Affiliations:** 1 Department of Environmental and Occupational Medicine, National Taiwan University College of Medicine, #1, Ren-Ai Rd. Sec. 1, Taipei, 10051 Taiwan; 2 Department of Environmental and Occupational Medicine, National Taiwan University Hospital, #7, Chung-Shan South Road, Taipei, 10002 Taiwan; 3 Department of Physical Medicine and Rehabilitation, National Taiwan University College of Medicine, #1, Ren-Ai Rd. Sec. 1, Taipei, 10051 Taiwan; 4 Department of Physical Medicine and Rehabilitation, National Taiwan University Hospital, #7, Chung-Shan South Road, Taipei, 10002 Taiwan

**Keywords:** Shoulder, Work-related, Repetition, Posture

## Abstract

**Background:**

The electronics industry is one of the largest global industries, and significant numbers of workers are engaged in this industry. Evidence suggests two associations, including one between ergonomic risks and shoulder disorders and another between psychological stress and psychological problems among workers in this industry. Investigations on ergonomic risks, psychological stress, and sex effects for shoulder disorders in this industry are limited. This study aimed to explore personal and work-related factors associated with shoulder disorders and to investigate the combined effect of similar ergonomic risk factors.

**Methods:**

In this cross-sectional study, 931 workers aged 20 to 58 from an electronics factory in Taiwan were recruited. A Nordic musculoskeletal questionnaire was used to assess shoulder symptoms. Sociodemographic factors and work-related factors, including psychological stress, were assessed. One hundred random sample workers with shoulder symptoms underwent a standardized clinical test for the evaluation of subacromial impingement syndrome. The ergonomic risks were assessed by the risk filter of ‘upper limb disorders in the workplace’, including repetition, posture, force, vibration, and duration of exposure.

**Results:**

The prevalence of shoulder symptoms was 30.5, and 19% of those with shoulder symptoms had subacromial impingement syndrome. In multivariable analyses, older age (adjusted odds ratio (aOR) = 1.37, 95% CI 1.01–1.86), repetition (aOR = 1.73, 95% CI 1.15–2.60) and posture (aOR = 1.85, 95% CI 1.10–3.11) were associated with shoulder symptoms. Regarding the gender effect, older age (aOR = 1.46, 95% CI 1.01–2.11), repetition (aOR = 1.64, 95% CI 1.00–2.68), posture (aOR = 1.89, 95% CI 1.01–3.52), and force (aOR = 1.68, 95% CI 0.99–2.85) were associated with shoulder symptoms in men, whereas posture (aOR = 2.12, 95% CI 0.99–4.57) was associated with symptoms in women.

**Conclusions:**

This study implies that repetition and posture are important risk factors for shoulder disorders in the electronics industry. The risk exhibited sex differences, and force was more important for shoulder disorders in men. Such information is useful to help occupational health practitioners and policy makers conduct preventive programmes on shoulder disorders in this working population. Future longitudinal studies on work-related shoulder disorders are warranted.

**Supplementary Information:**

The online version contains supplementary material available at 10.1186/s12889-021-11572-4.

## Background

The electronics industry was estimated to engage 18 million workers worldwide in 2010 [1] and is the leading industry in many East Asian countries, including Japan, South Korea, and Taiwan. Over 800,000 employers belonged to the industry in Taiwan in 2016 [2]. In addition to chemical and physical hazards, the work environment of the industry may contain ergonomic hazards, such as repetition, lifting, and awkward posture [3–6], making the population vulnerable to developing musculoskeletal disorders.

The manufacturing of thin film transistor liquid crystal displays (TFT-LCDs) is a common example of the electronics industry. Three processes occur during the manufacturing of TFT-LCD panels: array, cell, and module assembly processes. The array process is similar to the semiconductor manufacturing process [7], which has been linked to upper limb musculoskeletal symptoms. A dose-response effect between the symptoms and working hours was observed [8]. Next, the cell process joins the arrayed substrate to the colour-filter substrate; then, the space between two substrates is filled with liquid crystal. Finally, the module assembly process requires assembling components, such as circuits and backlight units, into the glass panel. In the module department of a TFT-LCD factory, Lu et al. found that the most prevalent location of musculoskeletal symptoms was the shoulder (59.8%) [9]. They also indicated that a high work-related ergonomic risk for the shoulder area was associated with the following factors: poor arm support, mismatched workstation design, and worker anthropometry. In another similar study of an electronic assembly factory, Pullopdissakul et al. [10] assessed four work-related ergonomic hazards, including repetitive motion, high force, awkward posture, and contact stress. They found that ergonomic hazards were associated with upper limb musculoskeletal disorders. Furthermore, the exposure profile in settings of the electronics industry may be characterized by combined ergonomic exposures. Although several studies have assessed the combined effect of ergonomic risks for musculoskeletal disorders [11–13], investigations on whether the combination of two or more similar ergonomic risks increases the risk of shoulder disorders are rarely addressed in the electronics industry. For example, workers exposed to both awkward joint positions and joints held in fixed positions have high odds of shoulder disorders compared to those with only one exposure.

Regarding the work environment, the manufacturing process of microelectronic products requires the protection of special work environments, namely, clean rooms, where employees need to be completely covered in protective suites [14]. When the workers remain completely suited while performing repetitive tasks during the entire work shift, these head-to-toe garments can cause discomfort and limit the range of body movements. Furthermore, the electronics industry is known for its rapid technological innovation, global competition, operation on shift work, and performance-based pay systems [3, 15]. The association between psychological stress and psychological problems in the industry has been identified [6], but very few studies have investigated the psychosocial risk factors for shoulder disorders.

Although current evidence suggests that personal factors (i.e., age and sex) [16–19], ergonomic risks [20, 21], and psychological stress [16, 22] were associated with shoulder disorders, there have been very few investigations on all three factors for shoulder disorders in the electronics industry. An understanding of modifiable risk factors is critical to facilitating future efforts to prevent shoulder disorders in the industry. Therefore, the first objective of this study was to explore potential work-related and personal factors among workers with shoulder disorders in a representative TFT-LCD factory. The second objective was to examine the combined effect of similar ergonomic risk factors for shoulder disorders.

## Methods

### Study population

**Fig. 1:**
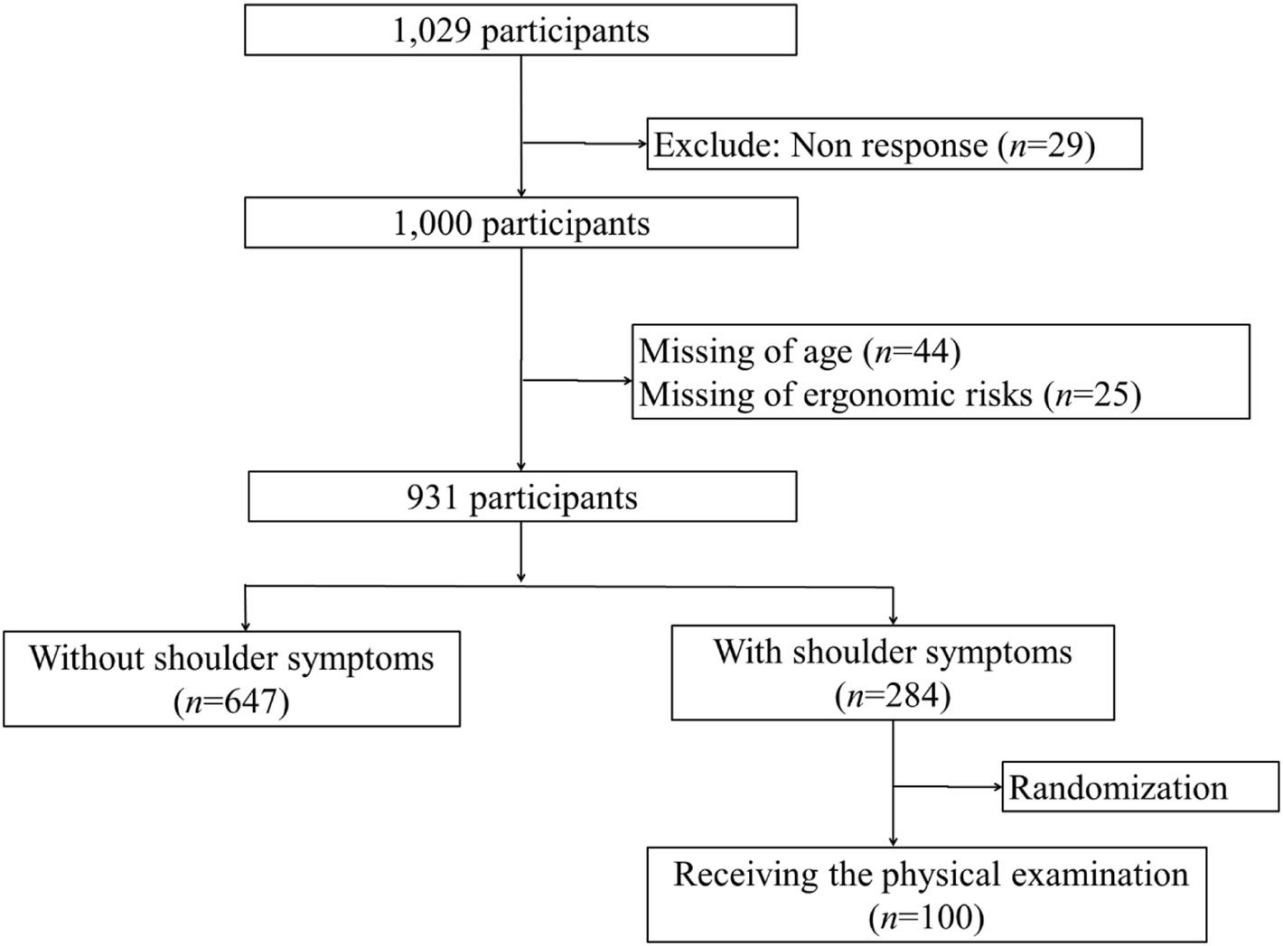
Flowchart of recruiting study population

This cross-sectional study was carried out in the following situation and process. In the period of the annual medical examination of an electronics enterprise in 2010, the formal written instructions for the study was posted at the place where the medical examination was performed. All available participants of the enterprise were invited to participate in the study, and a research staff member explained the detailed information about the study purpose, process, and their rights to the interested participants. The participants were given the opportunity to decline participation or to withdraw at any time. Privacy was guaranteed during the study, and informed consent was obtained from all individual participants recruited in the study. It was one of the largest TFT-LCDs in Taiwan with more than 1000 workers. Participants were recruited from the electronics enterprise. The inclusion criteria were age older than 20 years and working in the enterprise. A total of 1029 workers were eligible, and their age was between 20 and 58 years. Participants invited in the study were the population who received an annual medical examination, and one of the requirements for the examination was having work experience of more than half a year. Therefore, another duration of work experience was not considered for inclusion criteria. The exclusion criteria were foreign nationals in Taiwan. Based on a previous study of shoulder pain in a working population [23], an adjusted odds ratio = 1.73 and a one-year cumulative incidence rate = 6.6% were used to calculate the sample size. The calculation showed that a total of 655 patients would be required at a significance level of 0.05 and 95% power. A total of 1029 eligible participants were provided with information on the study, such as study purpose and methods. Twenty-nine participants refused to join the study (response rate: 97.2%). The final sample included 931 participants after excluding those with missing values for age (*n* = 44) and ergonomic risks (*n* = 25) (Fig. 1). In the study population, 13 participants had acute musculoskeletal disorders (such as driving accidents, falls, etc.).

### Outcome measures

The definition of shoulder symptoms within 12 months preceding the survey was based on the Nordic questionnaire [24]. The questionnaire of detailed shoulder symptoms on occupational cause, duration, frequency and severity of shoulder symptoms consisted of four items. The following items were included in the questionnaire: (1) Are your shoulder symptoms the occupational cause? (2) Have your shoulder symptoms lasted for more than 1 month during the last 12 months? (3) Are the frequency of your shoulder symptoms occurring more than once per week during the last 12 months? (4) Have your shoulder symptoms caused you to reduce your activity during the last 12 months? (Supplementary file 1) To understand the clinical diagnosis of shoulder symptoms, objective indicators based on special physical examinations of the shoulder were applied in this study. Physical examination was performed by an occupational physician using a standardized clinical procedure. The procedure strictly followed the clinical tests of the European consensus criteria document for the evaluation of the work-relatedness of upper extremity musculoskeletal disorders [25]. In the study, participants with shoulder symptoms were randomly assigned to a group (*n* = 100) receiving physical examination. The type of randomization was a simple randomization. Sequence generation was performed according to a computer-generated list of random numbers. Moreover, the jobs of workers with shoulder symptoms were not adjusted, such as changing to lighter duties.

### Assessment of work-related ergonomic risk factors

The assessment of work-related ergonomic risk factors was based on the risk filter of ‘upper limb disorders in the workplace’ issued by the Health and Safety Executive, UK [26], which is a technique for assessing exposure to risk factors for work-related upper limb musculoskeletal disorders. The main feature is a checklist for upper limb disorder hazards in the workplace to assess the four categories of ergonomic risk factors, movement frequency, posture, load/force, vibration, and consider the duration of exposure. This method is a simpler observational method that has the benefits of being low-priced and practical for use in the workplace and appears to offer the levels of generality and exactness matched to the needs of occupational safety and health practitioners [27]. There were 3 items about repetition-related risks, 6 items about posture-related risks, 6 items about force-related risks, and one item assessing vibration-related risks. Picture forms of different postures were used to facilitate participants’ understanding. A detailed description and definition are presented in Table 1. Furthermore, the definition of the duration of exposure for repetition, posture, and force was greater than 2 h per shift, and the definition for vibration was regular with some point during most shifts. The process of the assessment of work-related ergonomic risk factors contained three stages: (1) formal written instructions for the assessment of ergonomic risk factors edited by an occupational hygienist were posted in the workplace 1 month before assessment; (2) the occupational hygienist presented a verbal briefing based on a written script to the participants prior to the distribution of the assessment list; and (3) the occupational hygienist assessed any missing items of the assessment list, and these items were revised after discussing with the participants. To assess the combined effect of similar ergonomic risks, the individual risks for the categories of repetition, posture, and force were used to stratify the participants into three groups, including the high-risk (≥ 3 items), low-risk (1–2 items), and no-risk (no item) groups, with the exception of the category of vibration.

**Table 1:** Checklist of work-related ergonomic risk factors

**Repetition**	
Repeating the same motions every few seconds	
A sequence of movements repeated more than twice per minute	
More than 50% of the cycle time involved in performing the same sequence of motions	
**Postures**	
Large range of joint movement such as side to side or up and down	
Awkward or extreme joint positions	
Joints held in fixed positions	
Stretching to reach items or controls	
Twisting or rotating items or controls	
Working overhead	
**Force**	
Pushing, pulling, moving things (including with the fingers or thumb)	
Grasping / gripping	
Pinch grips i.e. holding or grasping objects between thumb and finger	
Steadying or supporting items or work pieces	
Shock and /or impact being transmitted to the body from tools or equipment	
Objects creating localized pressure on any part of the upper limb	
**Vibration**	
Use any powered hand-held or hand-guided tools or equipment / hand-feed work pieces to vibrating equipment	

### Assessment of associated variables

A structured self-administered questionnaire was distributed to collect the data, and the questions included the following aspects: (1) personal factors, including basic demographic information (i.e., age and sex), and body mass index; (2) work-related factors, including seniority, psychological stress, and work-related physical fatigue. Regarding the assessment of psychological stress, because the industry is highly globally competitive and requires a high level of information security, it would be impossible to administer detailed psychological stress assessments, such as job demand-control models and effort-reward imbalance models. Therefore, a single-item question was used as a surrogate and developed from the need to indicate stress at work based on the previous psychological stress measure [28, 29]. The definition of stress was feeling irritable, anxious, or having sleep problems as a consequence of work-related issues. The participants were asked to report the frequency of stress at work, and the response options were a four-point categorical scale: (1) never; (2) some periods; (3) several periods; and (4) permanent stress. The assessment of work-related physical fatigue was based on the method of Skarpsno et al. [30]. Participants were asked, ‘Is your work so physically demanding that you are often physically worn out after a day’s work?’. The response options were ‘never, or almost never’, ‘seldom’, ‘quite often’, and ‘yes, nearly always’ (Supplementary file 1).

### Statistical analysis

We examined the baseline characteristics, including age, sex, body height, body weight, body mass index, psychological stress, work-related physical fatigue, and ergonomic risk factors, among the participants. The baseline characteristics and ergonomic risk factors among workers with subacromial impingement syndrome confirmed by physical examination were examined. The descriptive results of continuous variables are expressed as the mean (standard deviation), and the categorical variables are presented as numbers and percentages. The continuous and categorical data were compared between the participants with and without shoulder symptoms. For categorical data, Chi-squared tests was used. The Shapiro-Wilk test was applied to test the normal distribution for continuous data, including body height, body weight, body mass index, and experience at the job. The Wilcoxon rank-sum test was applied for nonnormal distribution. Univariable logistic regression was used to identify factors associated with shoulder symptoms. The variables considered in the analysis included age, sex, body mass index, psychological stress, work-related physical fatigue, repetition, posture, force, and vibration. Regarding ergonomic risk factors, the high- or low-risk groups were compared with the no risk group. Multivariable logistic regression analysis was performed to adjust for variables exhibiting significant associations in the univariable analysis, and all variables significant in univariate analysis were included in the model. Multivariable regression analysis was performed again for the population excluding participants with acute musculoskeletal disorders. For multivariable logistic regression analysis, interactions between significant explanatory variables were tested. To understand shoulder symptoms with occupational cause, we applied the four items of the questionnaire, including occupational causes, duration, frequency and severity of symptoms, to define the occupational shoulder symptoms. The above analyses were performed again. Moreover, the present study applied sex-stratified analyses rather than sex-adjusted analyses as recommended by Silverstein et al. [16] to better understand the potential sex differences in the risk for shoulder symptoms. A *p*-value of < 0.05 was considered to indicate a statistically significant difference. All analyses were performed using SAS software version 9.4 (SAS Institute, Cary, North Carolina).

## Results

The basic characteristics of the study population are presented in Table 2. We recruited 931 participants, including 284 workers (30.5%) with shoulder symptoms and 647 workers (69.5%) without shoulder symptoms. Although the mean ages (standard deviation) were 38.3 (7.0) and 37.4 (7.4) years for people with and without shoulder symptoms, respectively (*p* = 0.10), a higher proportion of people with shoulder symptoms were over 40 years of age compared with those with no symptoms (*p* = 0.04). More workers were men in both groups, but the proportion of women with shoulder symptoms was significantly increased compared with those with no symptoms (*p* = 0.03). The difference in psychological stress between the two groups was not significant (*p* = 0.14), and the difference in work-related physical fatigue was also not significant (*p* = 0.14). For workers with occupational shoulder symptoms, psychological stress and work-related physical fatigue were significantly associated with their symptoms (*p* = 0.03 and < 0.01, respectively) (Supplementary Table 1). Regarding the results of the physical examination of shoulders, a random sample of 100 workers (35.2%) selected from a total of 284 workers with shoulder symptoms received the examination. Among them, 19.0% had subacromial impingement syndrome confirmed by physical examination. The baseline characteristics and ergonomic risk factors among workers with subacromial impingement syndrome were presented in Supplementary Tables 2 and 3.

**Table 2:** Basic characteristics of study population and distribution of shoulder symptoms

Variables	Shoulder symptoms		No shoulder symptoms		
	*n* = 284		*n* = 647		p-value
Age (years)					0.04
≦40	174	(61.3%)	442	(68.3%)	
> 40	110	(38.7%)	205	(31.7%)	
Sex					0.03
Female	96	(33.8%)	174	(26.9%)	
Male	188	(66.2%)	473	(73.1%)	
Body height (cm)	167.0	(8.8)	167.4	(8.3)	0.39
Body weight (Kg)	68.8	(14.8)	70.0	(13.6)	0.06
Body mass index (kg/m^2^)	24.5	(4.1)	24.9	(4.1)	0.13
Experience at the job (years)	5.2	(5.1)	5.3	(5.6)	0.69
Psychological stress^a^					0.14
Never	24	(8.5%)	72	(11.1%)	
Some periods	120	(42.3%)	274	(42.4%)	
Several periods	112	(39.4%)	262	(40.5%)	
Permanent	28	(9.9%)	39	(6.0%)	
Work-related physical fatigue^a^					0.06
Never or almost never	15	(5.3%)	52	(8.0%)	
Seldom	101	(35.6%)	272	(42.0)	
Quite often	143	(50.4%)	279	(43.1%)	
Yes, nearly always	25	(8.8%)	44	(6.8%)	

Data are presented as number (%), mean (SD). ^a^The sum of percentage were not 100% due to round off to the first decimal place

**Fig. 2:**
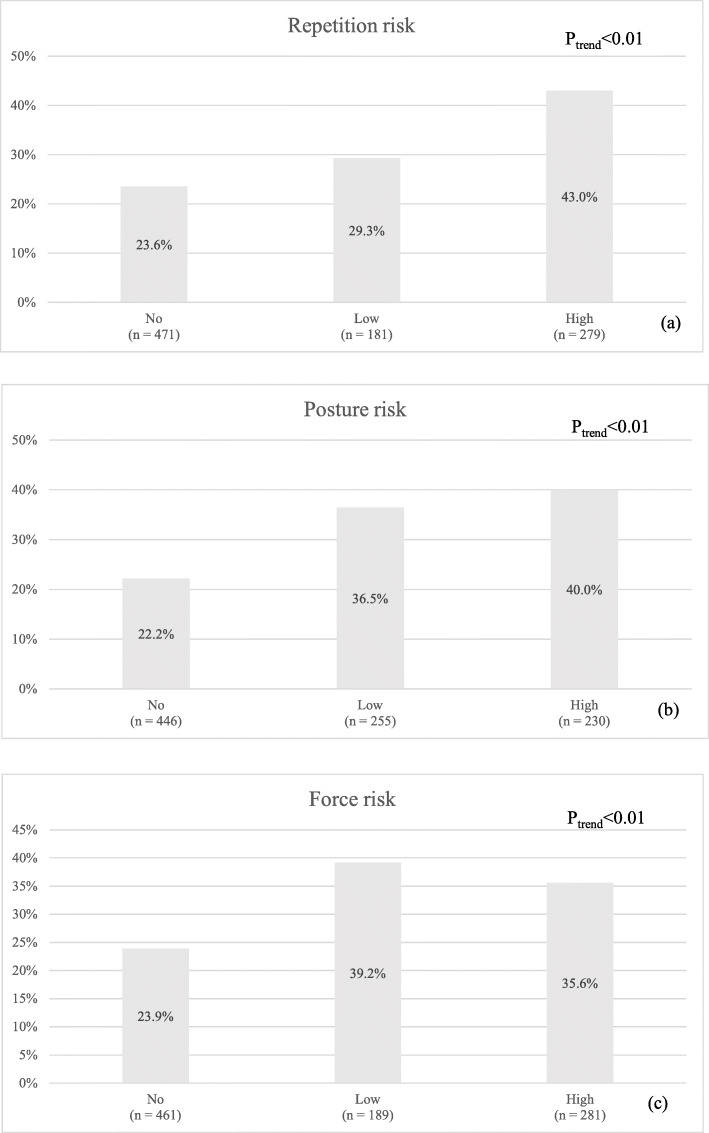
Association between the proportions of shoulder symptoms and the number of ergonomic risks: (**a**) Repetition risk, (**b**) Posture risk, (**c**) Force risk. Footnotes: The definition of high, low, and no were ≥ 3 items, 1–2 items, and no item of repetition, posture, and force risks, respectively; P_trend_: P for trend

Ergonomic risk factors for workers with and without shoulder symptoms are presented in Table 3. Compared with the no symptoms group, the group with shoulder symptoms had significantly higher rates of repetition risks, including working with repeating the same motion every few seconds, performing a sequence over twice per minute, and over half of the cycle time in the same sequence of motions (all *p*-values < 0.01). For the risks related to working postures, the group with shoulder symptoms exhibited significantly higher rates of working with awkward/extreme joint positions, joints held in fixed positions, stretching to reach items, twisting/rotating items, and working overhead (all *p*-values < 0.01). For force-related risk, the group with shoulder symptoms had significantly higher rates of working with pushing/pulling/moving things, grasping/gripping, pinch grips, shock/impact being transmitted to the body, and localizing pressure on the upper limb (*p* ≤ 0.03). There was no significant difference in the proportions of workers using vibrating equipment between the groups with/without shoulder symptoms (*p* = 0.24). For workers with occupational shoulder symptoms, all items of repetition, all items of posture, and most items of force risks were associated with their symptoms (Supplementary Table 4). Furthermore, the combined effect of similar ergonomic risks for the proportion of shoulder symptoms is shown in Fig. 2. For the three different risk groups, the group with high combined repetition risks had higher proportions of shoulder symptoms than the group with low risks (P for trend < 0.01), and similar trends were found for the posture and force risks (two P for trend < 0.01).

**Table 3:** Distribution of biomechanical risks for shoulder symptoms

	Shoulder symptoms		No shoulder symptoms		
Variables	n = 284		n = 647		*p*-value
**Repetition risk**					
Repeating the same motions every few seconds	135	(47.5%)	199	(30.8%)	< 0.01
A sequence of movements repeated more than twice per minute	146	(51.4%)	220	(34.0%)	< 0.01
More than 50% of the cycle time involved in performing the same sequence of motions	157	(55.3%)	249	(38.5%)	< 0.01
**Posture risk**					
Large range of joint movement such as side to side or up and down	59	(20.8%)	107	(16.5%)	0.12
Awkward or extreme joint positions	53	(18.7%)	54	(8.3%)	< 0.01
Joints held in fixed positions	126	(44.4%)	169	(26.1%)	< 0.01
Stretching to reach items or controls	115	(40.5%)	201	(31.1%)	< 0.01
Twisting or rotating items or controls	113	(39.8%)	177	(27.4%)	< 0.01
Working overhead	59	(20.8%)	90	(13.9%)	< 0.01
**Force risk**					
Pushing, pulling, moving things (including with the fingers or thumb)	140	(49.3%)	244	(37.7%)	< 0.01
Grasping/gripping	146	(51.4%)	240	(37.1%)	< 0.01
Pinch grips i.e. holding or grasping objects between thumb and finger	98	(34.5%)	177	(27.4%)	0.03
Steadying or supporting items or work pieces	78	(27.5%)	142	(21.9%)	0.07
Shock and/or impact being transmitted to the body from tools or equipment	35	(12.3%)	50	(7.7%)	0.03
Objects creating localized pressure on any part of the upper limb	50	(17.6%)	68	(10.5%)	< 0.01
**Vibration risk**					
Use any powered hand-held or hand-guided tools or equipment/ hand-feed work pieces to vibrating equipment	29	(10.2%)	51	(7.9%)	0.24

The univariable and multivariable-adjusted odds ratios for the associations between the risk factors and shoulder symptoms are presented in Table 4. Older age (odds ratio (OR) = 1.36, 95% confidence interval (95% CI) = 1.02–1.82, using age ≦ 40 as the reference) and female sex (OR = 1.39, 95% CI = 1.03–1.88, using males as the reference) were significantly associated with shoulder symptoms. Permanent psychological stress was significantly associated (OR = 2.15, 95% CI = 1.10–4.21, using never stress as the reference). Among the ergonomic risk factors, high repetition-related risk (OR = 2.45, 95% CI = 1.78–3.37, using no repetition risk as the reference) was significantly associated with shoulder symptoms. Low and high posture-related risks (OR = 2.01, 95% CI = 1.43–2.82; OR = 2.34, 95% CI = 1.65–3.30, using no posture risk as the reference) were significantly associated with shoulder symptoms. Low and high force-related risks (OR = 2.05, 95% CI =1.43–2.95; OR = 1.76, 95% CI = 1.27–2.44, using no force risk as the reference) were significantly associated with shoulder symptoms. Regarding the multiple regression analysis, no model selection was applied to the model. The findings showed that older age (OR = 1.37, 95% CI = 1.01–1.86, using age ≦ 40 as the reference) was significantly associated with shoulder symptoms. High repetition-related risk (OR = 1.73, 95% CI = 1.15–2.60, using no repetition risk as the reference) and low and high posture-related risks (OR = 1.61, 95% CI = 1.06–2.45; OR = 1.85, 95% CI = 1.10–3.11, using no posture risk as the reference) were significantly related to shoulder symptoms. For the population excluding participants with acute musculoskeletal disorders, the findings showed that older age (OR = 1.37, 95% CI = 1.01–1.85, using age ≦ 40 as the reference) was significantly associated with shoulder symptoms. High repetition-related risk (OR = 1.72, 95% CI = 1.14–2.59 using no repetition risk as the reference) and low and high posture-related risks (OR = 1.62, 95% CI = 1.06–2.46; OR = 1.88, 95% CI = 1.12–3.18, using no posture risk as the reference) were significantly related to shoulder symptoms. Moreover, the interaction between repetition and posture in the model was tested, and its result was not significant (*p*-value = 0.33). No interaction was noted between age and repetition or age and posture in the model (*p*-value = 0.18 and 0.52, respectively). In the model, sex, body mass index, psychological stress, work-related physical fatigue, force risk, and vibration were not significantly associated with shoulder symptoms. For workers with occupational shoulder symptoms, repetition and posture risks were associated with their symptoms in the regression model (Supplementary Table 5).

**Table 4:** Univariate and multivariate logistic regression analysis of factors influencing shoulder symptoms

	Shoulder symptoms		No shoulder symptoms		Univariate analysis			Multivariate model^d^		
	n	%	n	%	OR	95%CI	p-value	OR	95%CI	*p*-value
Age (years)							0.04	1.37	1.01–1.86^a^	0.04
> 40^e^					1	–				
≦40					1.36	1.02–1.82^a^				
Sex							0.03	1.03	0.74–1.43	0.85
Male^e^					1	–				
Female					1.39	1.03–1.88^a^				
Body mass index (kg/m^2^)					0.98	0.94–1.01	0.18			
Psychological stress							0.15			0.30
Never^e^					1	–		1	–	
Some periods					1.31	0.79–2.19		1.28	0.76–2.16	
Several periods					1.28	0.77–2.14		1.21	0.72–2.06	
Permanent					2.15	1.10–4.21^a^		1.91	0.95–3.84	
Work-related physical fatigue							0.06			
Never or almost never^e^					1	–				
Seldom					1.29	0.69–2.39				
Quite often					1.78	0.97–3.27				
Yes, nearly always					1.97	0.93–4.19				
Repetition risk							< 0.01			< 0.01
No^e^	111	39.1	360	55.6	1	–		1	–	
Low	53	18.7	128	19.8	1.34	0.91–1.97		0.97	0.63–1.51	
High	120	42.3	159	24.6	2.45	1.78–3.37^c^		1.73	1.15–2.60^b^	
Posture risk							< 0.01			0.04
No^e^	99	34.9	347	53.6	1	–		1	–	
Low	93	32.8	162	25.0	2.01	1.43–2.82^c^		1.61	1.06–2.45^a^	
High	92	32.4	138	21.3	2.34	1.65–3.30^c^		1.85	1.10–3.11^a^	
Force risk							< 0.01			0.08
No^e^	110	38.7	351	54.3	1	–		1	–	
Low	74	26.1	115	17.8	2.05	1.43–2.95^c^		1.38	0.91–2.12	
High	100	35.2	181	28.0	1.76	1.27–2.44^b^		0.86	0.54–1.38	
Vibration risk							0.24			
No^e^	255	89.8	596	92.1	1	–				
Yes	29	10.2	51	7.9	1.33	0.82–2.15				

*OR* odds ratio; *CI* confidence interval; ^a^*p* < 0.05, ^b^*p* < 0.01, ^c^*p* < 0.0001; ^d^Multivariable model included: independence of risk factors in the univariate analysis and no model selection; ^e^Reference group

To identify possible sex-specific factors, the population was stratified by sex, and the results are shown in Table 5. Univariable regression analysis showed that older age, using age ≦ 40 as the reference, was significantly associated with shoulder symptoms only in men and not in women. High repetition, low and high posture, and low and high force risks were significantly associated with shoulder symptoms in men. High repetition and low and high posture risks were significantly associated with shoulder symptoms in women. Body mass index and vibration were not significantly associated with shoulder symptoms in men and women. Psychological stress and work-related physical fatigue were significantly associated in women. Regarding the multiple regression analysis for men, the findings showed that high repetition (OR = 1.64, 95% CI = 1.00–2.68) and high posture (OR = 1.89, 95% CI = 1.01–3.52) were significantly associated with shoulder symptoms. Force was approximately significantly associated with shoulder symptoms (*p* = 0.05; OR = 1.68, 95% CI = 0.99–2.85). In the model for women, the findings showed that posture was approximately significantly associated with shoulder symptoms (p = 0.05; OR = 2.12, 95% CI = 0.99–4.57). Furthermore, a lower value in the higher category of force-related risks among the overall population (Table 4), force-related risks among men workers (Table 5), and posture-related risks among women (Table 5) may be an indication of a healthy worker effect.

**Table 5:** Univariate and multivariate logistic regression analysis of factors influencing shoulder symptoms, stratified by sex

	Men										Women									
	Shoulder symptoms		No shoulder symptoms		Univariate analysis			Multivariate model^d^			Shoulder symptoms		No shoulder symptoms		Univariate analysis		Multivariate model^d^			
	n	%	n	%	OR	95%CI	p-value	OR	95%CI	p-value	n	%	n	%	OR	95%CI	p-value	OR	95%CI	p-value
Age (years)							0.04	1.46	1.01–2.11^a^	0.05					1.1	0.66–1.81	0.72			
> 40^e^					1	–														
≦40					1.45	1.01–2.07^a^														
Body mass index (kg/m^2^)					1	0.96–1.05	0.99								0.95	0.90–1.01	0.10			
Psychological stress							0.86										0.02			0.16
Never^e^					1	–									1	–		1	–	
Some periods					1.11	0.59–2.08									1.82	0.76–4.36		1.15	0.33–3.96	
Several periods					1.16	0.62–2.18									1.63	0.66–4.01		0.72	0.18–2.88	
Permanent					1.40	0.62–3.15									8.94	2.22–35.95^b^		5.50	0.55–54.66	
Work-related physical fatigue							0.24										0.06			0.49
Never or almost never^e^					1	–									1	–		1	–	
Seldom					1.01	0.47–2.17									2.00	0.70–5.69		2.02	0.48–8.58	
Quite often					1.46	0.69–3.09									2.80	0.98–8.04^c^		2.94	0.61–14.24	
Yes, nearly always					1.24	0.48–3.19									5.13	1.38–19.11^a^		1.53	0.17–14.22	
Repetition risk							< 0.01			0.09							< 0.01			0.12
No^e^	89	13.5	291	61.5	1	–		1	–		22	22.9	69	39.7	1	–		1	–	
Low	40	21.3	93	19.7	1.41	0.91–2.18		1.02	0.62–1.69		13	13.5	35	20.1	1.17	0.53–2.59		0.84	0.33–2.10	
High	59	31.4	89	18.8	2.17	1.45–3.25^b^		1.64	1.00–2.68^a^		61	63.5	70	40.2	2.73	1.52–4.93^b^		1.71	0.79–3.70	
Posture risk							< 0.01			0.14							< 0.01			0.14
No^e^	78	41.5	276	58.4	1	–		1	–		21	21.9	71	40.8	1	–		1	–	
Low	58	30.9	115	24.3	1.78	1.19–2.67^b^		1.37	0.83–2.26		35	36.5	47	27.0	2.52	1.31–4.85^b^		2.12	0.99–4.57^c^	
High	52	27.7	82	17.3	2.24	1.46–3.45^b^		1.89	1.01–3.52^a^		40	41.7	56	32.2	2.42	1.28–4.55^b^		1.52	0.66–3.51	
Force risk							< 0.01			0.06							0.27			
No^e^	84	44.7	287	60.7	1	–		1	–		26	27.1	64	36.8	1	–				
Low	45	23.9	69	14.6	2.23	1.43–3.49^b^		1.68	0.99–2.85^c^		29	30.2	46	26.4	1.55	0.81–2.98				
High	59	31.4	117	24.7	1.72	1.16–2.56^b^		0.92	0.52–1.64		41	42.7	64	36.8	1.58	0.86–2.88				
Vibration risk							0.22										0.89			
No^e^	170	90.4	441	93.2	1	–					85	88.5	155	89.1	1	–				
Yes	18	9.6	32	6.8	1.46	0.80–2.67					11	11.5	19	10.9	1.06	0.48–2.32				

*OR* odds ratio; *CI* confidence interval; ^a^*p* < 0.05, ^b^*p* < 0.01, ^c^*p* = 0.05(borderline significant); ^d^Multivariable model included: independence of risk factors in the univariate analysis and no model selection; ^e^Reference group

## Discussions

The present study explored the work-related and personal factors among a special working population, namely, TFT-LCD factory workers. The multiple regression model showed that older age, repetition, and posture were associated with shoulder symptoms; however, psychological stress and work-related physical fatigue were not associated with shoulder symptoms. The results were similar to the population excluding participants with acute musculoskeletal disorders. Workers with more repetition, posture, or force risks reported a higher proportion of shoulder symptoms (Fig. 2.), and these findings support the combined effect of similar ergonomic risks for shoulder disorders. This combined effect approach was similar to the ‘Key Indicator Method for Manual Handling Operations’ and combines the main risk factors for force, repetition, posture, and others into a single risk score [31]. Furthermore, we identified sex differences in the effect of exposure to the risk factors for shoulder symptoms. For men, older age, repetition, posture, and force were associated with shoulder symptoms. For women, posture was associated with shoulder symptoms. This finding implies that force was more important for shoulder disorders in men. Furthermore, permanent psychological stress was associated with shoulder symptoms in univariable regression, but the finding in the regression model was not found after considering other factors, such as ergonomic risks. To the best of our knowledge, this is the first study to investigate shoulder symptoms and physical examination of subacromial impingement syndrome accompanied by potential personal factors, ergonomic risks, and psychological stress among workers in an electronics factory.

The ergonomic risks or musculoskeletal disorders among workers in the TFT-LCD industry are rarely addressed. Only two studies indicated that high ergonomic risks for the shoulder area were associated with poor arm support and the discrepancy between the workstation and the workers’ anthropometry [9, 32]. No comprehensive analysis has considered different ergonomic risks (e.g., repetition, posture, force, and vibration) as well as personal factors and psychological stress for shoulder disorders. Nevertheless, studies in the semiconductor industry, which shares similar work procedures with those of the TFT-LCD industry, have indicated that shoulder symptoms are among the most prevalent musculoskeletal disorders [33–35]. In one of the earliest studies in 1986, Kilbom et al. indicated that flexion and abduction of the upper arm were associated with shoulder symptoms [33]. Chandrasakaran et al. showed that prolonged sitting and trunk bending were associated with shoulder symptoms [36]. Chee et al. indicated that prolonged sitting in awkward postures with the characteristics of a forward bent neck and tables that are too high may result in shoulder pain [7]. Furthermore, due to the high accuracy requirements for tasks, such as inspection or manual assembly, workers tend to bend their necks forward to give optimal visual conditions and could cause shoulder symptoms [37]. Aghilinejad et al. found that the use of magnification loupes may improve the visibility of electronic parts as well as improve the postures of assembly workers and may reduce musculoskeletal discomfort [35]. The aforementioned findings were similar to those of the present study, which demonstrated that posture was an independent ergonomic risk for shoulder symptoms in the two regression models, indicating that awkward or extreme joint positions, joints held in fixed positions, stretching to reach items or controls, and working overhead were significant risk factors. The possible reasons accounting for the association between posture and shoulder symptoms are that arm elevation or prolonged sitting with awkward posture (e.g., bent neck) may place additional load on the musculoskeletal system of the shoulder. One of the main pathophysiological mechanisms of shoulder disorder (e.g., subacromial impingement syndrome) is compression of the tendons between the humeral head and the coracoacromial arch and ischaemia by impingement or increased intramuscular pressure as a result of arm elevation [17].

Regarding the association between vibration and shoulder symptoms, the meta-analysis indicated low to very low evidence for an association between shoulder disorders and hand-arm vibration (OR = 1.3) [38]. In positive association studies, the study populations were special working populations, such as forestry workers, rock drill workers, construction workers, and railroad engineers [39–42], and the hospital served as the recruitment location [43]. Hagberg et al. indicated that the exposure factors associated with rotator cuff tendinitis in different occupational groups were not the same [44]. In the present study, an association was not found in the regression model, which was similar to earlier studies for electronics workers [8, 36]. It is possible that the difference in study populations or locations recruited may explain these inconsistent findings. A high prevalence of shoulder pain was found in the electronics industry, which could be related to repetitive lifting tasks, repetitive operating machines, and monotonous short cycles of tasks [7]. Chee et al. found that repetitive tasks could increase the risk of shoulder pain [45]. A longitudinal study in France indicated that repetitive work under time constraints contributed to the development of chronic neck and shoulder disorders after adjustment for age [22]. Furthermore, Jonsson et al. showed that reorganizing monotonous and repetitive work into a more diverse pattern may improve work-related upper limb musculoskeletal disorders after a 2-year follow-up study [34]. These findings among electronics workers are consistent with the present study, indicating that work that involves repeating the same motion every few seconds, a sequence of movements over twice per minute, and over half of the cycle time in the same sequence of motion were significant risk factors.

Regarding the association between force and shoulder symptoms, one systematic review revealed that shoulder load (OR = 2.0) and hand force exertion (OR = 1.5) were associated with shoulder disorders [38]. Another systematic review indicated that the occurrence of subacromial impingement syndrome was associated with high maximal voluntary contraction, lifting, and high hand force (OR = 2.8–4.2) [46]. Repetitive tasks using mechanical force that put stress on small areas increased the prevalence of neck or shoulder pain in the department of manual assembly in 18 electronics factories [8]. One possible mechanism accounting for the association between force and shoulder symptoms is that the direction of the force performed increases muscular activity levels, especially in overhead work [47]. Similar to the above studies, an association between force and shoulder symptoms was found (crude OR = 1.76–2.05) in the univariate analysis (Table 4), but an association was not found after the multiple regression. One possible reason accounting for the lack of association is that the production process is typically automated and the process changes to light objects in the electronics industry.

Regarding sex differences, the association between shoulder symptoms and the frequency of forceful exertions was higher for women than men in a sex-stratified analysis [48]. Women are considered to be at a higher risk of shoulder disorders (e.g., rotator cuff syndrome) than men, possibly reflecting both biological predisposition and exposure to work-related repetitive biomechanical constraints [16, 17]. The biological distinctions between men and women, including anatomy, strength, hormones, neuromuscular control, and musculoskeletal flexibility [49], suggest a different vulnerability to these work-related risk factors for shoulder disorder. An association between force and shoulder symptoms was not found for women in the two regression models (Table 5). The sex difference may result from differences in the type of task assigned, which means different exposures to the constraints at work [16, 17]. Women and men in the same industry may have different tasks, interactions between equipment and tool dimensions, and work activities [16]. In the present study, a majority of men workers (30.5%) were assigned to tasks that involved the handling of heavy objects, whereas fewer women workers (10.4%) were assigned to tasks that involved the handling of heavy objects. Therefore, it is possible that men workers had a higher opportunity of exposure to force risk than women. Furthermore, the results of the present study from the regression model found that repetition, posture and force (approximately significant) were risk factors for shoulder symptoms in men, whereas posture (approximately significant) was a risk factor in women (Table 5). Further investigation is needed to elucidate whether specific task assignments are associated with shoulder symptoms. Regarding posture and shoulder symptoms in women, earlier studies among women workers in electronics factories revealed that shoulder symptoms were the most common musculoskeletal disorders [33, 34]. Kilbom et al. indicated that flexion and abduction of the upper arm were associated with shoulder symptoms [33]. Miranda et al. found that the risk of chronic shoulder disorders was associated with working in awkward postures in women (adjusted OR = 2.3) [18]. The present findings in the regression model are consistent with earlier studies showing that posture (crude OR = 2.42–2.52; adjusted OR = 2.12, which was approximately significant) was associated with shoulder symptoms in women (Table 5).

The present study indicated that permanent psychological stress was a significant risk factor for shoulder symptoms in women based on univariable regression (Table 5). This finding is consistent with an earlier study that found that women may have jobs with higher psychosocial stress (e.g., high demands, low control), negatively impacting musculoskeletal health [16]. Although the biological pathway for shoulder disorders is biomechanical, psychological factors (e.g., work stress) may function as intermediating factors affecting these ergonomic risk factors [38]. Two possible reasons accounting for the association between psychological stress and shoulder symptoms for women are that the hardness of shoulder muscle for women is larger than that of men and that women are more sensitive to symptoms of their shoulder [50]. Furthermore, women reported higher levels of work overload, stress, and conflict compared with men due to the combined stress from the workplace and family (e.g., taking care of children) [51]. Women may accumulate risk factors related to work activities and activities of daily living; thus, high job demand contributes to the development of chronic neck and shoulder pain independently of age [22]. The association between psychological stress and shoulder symptoms in women was not significant after the multiple regression, and only posture were associated. The present findings are inconsistent with earlier studies. It is possible that the use of different methods to assess psychological stress could explain the different findings given that the single-item question on the frequency of stress at work was applied in the present study. Further investigation is needed to explore the issue using the different psychosocial stress models (e.g., job demand-control model, effort–reward imbalance model) for high-risk groups in the industry instead of the single-item question.

Age is a predictor for shoulder symptoms in earlier studies [18, 19]. The reason may be related to the pathophysiological mechanisms of increasing degeneration of the shoulder tendons and the development of osteoarthrosis in shoulder joints [17, 48]. Although ageing may play a role in shoulder symptoms in the working population, contradictory findings have been indicated among some working populations exposed to high biomechanical risks [17]. For example, Silverstein et al. indicated that age was marginally significant for shoulder disorders [52]. The present study found that age is a risk factor for shoulder symptoms only for men in the regression model possibly because the modification of the age effect was different for different sexes [17]. This present finding of examining subacromial impingement syndrome was similar to that of earlier studies that showed that subacromial impingement syndrome was a common cause of musculoskeletal pain in the general working population [12, 17, 52], and a correlation between shoulder symptoms and clinical signs of rotator cuff tendinitis by physical examination was identified [53].

Several limitations should be noted. First, this was a study in a single facility, and the generalizability of this study requires further assessment. Second, the cross-sectional design restricts the inference of causal relationships and can only determine the association between relevant risk factors and shoulder symptoms. Third, the multivariable logistic regression model did not consider other potential confounding variables for shoulder symptoms, such as lack of sufficient rest, work organization factors, physical activity in spare time, muscular endurance in the arms, and job satisfaction [19, 34, 54]. Fourth, the risk assessment tool used was mainly by self-administered questionnaire, and not by experts. The ergonomic risk factors analysed for repetition, posture, and force were only analysed for more than 2 h per shift; thus, one risk factor or combined risk factors with less this duration were not included. Moreover, the prolonged periods of exposure and working longer hours each day were not assessed. Thus, the results of the present study may be underestimated. Fifth, physical examination for subacromial impingement syndrome was only performed among workers with shoulder symptoms. The positive rate of subacromial impingement syndrome remains uncertain in workers without shoulder symptoms, and in the viewpoint of early prevention, further examination for non-shoulder symptom workers may explore subclinical shoulder cases. On the other hand, given that not all participants did receive a physical examination and shoulder symptoms served as outcomes, misclassification of health status may have occurred. Further investigation is needed to assess work-related shoulder disorders, such as integrating comprehensive exposure assessments into the intensity, duration, and frequency of ergonomic risk factors. This work may aid our understanding of the pathophysiological mechanisms of shoulder disorders as well as attributional fractions of relevant risk factors. Studies to improve the knowledge of sex and the physical and psychosocial aspects of job interactions could enhance workplace job design and policy on the prevention of work-related shoulder disorders.

## Conclusions

In the electronics industry, repetition and posture are important risk factors for shoulder disorders. The risk showed a sex difference, and force was more important for shoulder disorders in men than in women. Vibration is less important for shoulder disorders in the industry. A significant combined effect of similar ergonomic risks for shoulder disorders was noted. Therefore, future ergonomic risk assessments for shoulder disorders may include at least the following aspects: (1) personal factors; (2) repetition; (3) posture; and (4) psychological stress in the electronics industry. This information may be a useful reference in the working environment to help multifactorial intervention strategies reduce the risk of shoulder disorders. The development of a programme for the early detection and prevention of shoulder symptoms in this working environment is warranted. The sex difference may be taken into consideration for preventive strategies and relevant occupational health policies for shoulder disorders. Future large-scale studies with longitudinal follow-up are warranted to further elucidate the impacts of personal factors, ergonomic risks, and psychological stress on the shoulder disorders of workers in the electronics industry.

## Supplementary Information

**Additional file 1: **Questionnaire about shoulder symptom. This is the questionnaire about shoulder symptom developed in this study, including basic information, information on shoulder symptom, and work-related information. **Supplementary Table 1**: Basic characteristics of study population with and without subacromial impingement syndrome. **Supplementary Table 2**: Distribution of biomechanical risks for study population with and without subacromial impingement syndrome. **Supplementary Table 3**: Basic characteristics of study population and distribution of occupational shoulder symptoms. **Supplementary Table 4**: Distribution of biomechanical risks for occupational shoulder symptoms. **Supplementary Table 5**: Univariate and multivariate logistic regression analysis of factors influencing occupational shoulder symptoms.

## Data Availability

The dataset generated and/or analysed during the current study are available in the [Open Science Framework] repository, [https://osf.io/dm7xc/?view_only=bf198c5440b94cdfa859048328579077].

## Abbreviations

TFT-LCD: Thin film transistor liquid crystal display
OR: Odds ratios
aOR: Adjusted odds ratio
95% CI: 95% Confidence interval
