# Supplementary material for: Work-related and personal factors in shoulder disorders among electronics workers: findings from an electronics enterprise in Taiwan
Source: BMC Public Health. 2021 Aug 9;21:1525. doi: 10.1186/s12889-021-11572-4 (PMC8351339; doi:10.1186/s12889-021-11572-4)
Supplement: Supplementary file 1 — Additional file 1: Questionnaire about shoulder symptom. This is the questionnaire about shoulder symptom developed in this study, including basic information, information on shoulder symptom, and work-related information. Supplementary Table 1: Basic characteristics of study population with and without subacromial impingement syndrome. Supplementary Table 2: Distribution of biomechanical risks for study population with and without subacromial impingement syndrome. Supplementary Table 3: Basic characteristics of study population and distribution of occupational shoulder symptoms. Supplementary Table 4: Distribution of biomechanical risks for occupational shoulder symptoms. Supplementary Table 5: Univariate and multivariate logistic regression analysis of factors influencing occupational shoulder symptoms. [file 12889_2021_11572_MOESM1_ESM.zip › Questionnaire about shoulder symptomR4.docx]

**Questionnaire about shoulder symptom**

1. Basic information
   1. Date of inquiry: / /

year/month/day

- 1. What year were you born?
  2. Sex: □Female □Male
  3. How much do you weight? kg
  4. How tall are you? cm
  5. How many years and months have you been doing your present type of work?

Year month

1. Information on shoulder symptom
   1. In this picture you can see the approximate position of the shoulders, and please answer the following answers. The shoulder symptom is meant ache, pain or discomfort in the shaded area. Please concentrate on this area, ignoring any symptom you may have in adjacent parts of the body.


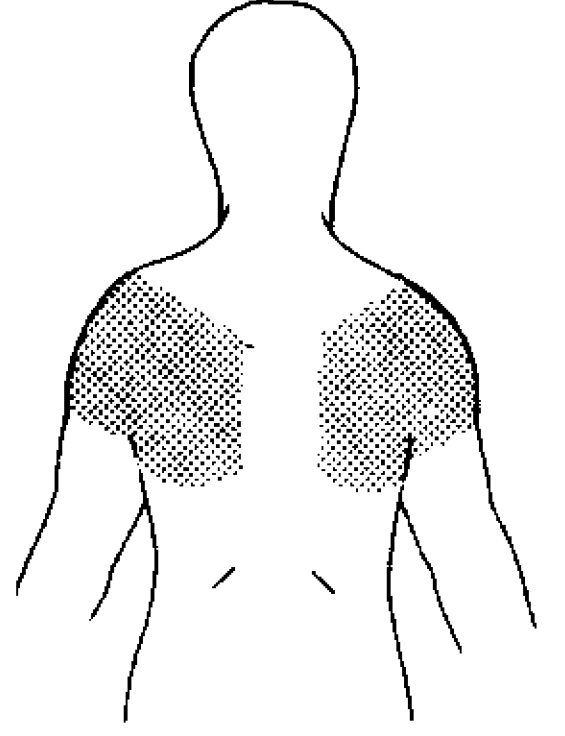


- 1. Have you ever hurt your shoulder in an accident (such as driving accidents, falls, etc.)?

□No □Yes, in the right shoulder

□Yes, in the left shoulder

□Yes, in both shoulders

- 1. Have you at any time during the last 12 months had symptoms in shoulders:

□No □Yes, in the right shoulder

□Yes, in the left shoulder

□Yes, in both shoulders

- 1. Have your shoulder symptoms lasted for more than one month during the last 12 months? □No □Yes
  2. Are the frequency of your shoulder symptoms occurring more than once per week during the last 12 months? □No □Yes
  3. Have your shoulder symptoms caused you to reduce your activity during the last 12 months? □No □Yes

1. Work-related information
   1. Are your shoulder symptoms the occupational cause? □No □Yes
   2. Over the past year, how often have you suffered from the stress at work?

(The stress at work is meant feeling irritable, anxious, or having sleep problems as a consequence of the work-related problem)

□never □some periods □several periods □permanent

- 1. Is your work so physically demanding that you are often physically worn out after a day’s work?

□never, or almost never □seldom □quite often □yes, nearly always
