# Supplementary material for: Work-related and personal factors in shoulder disorders among electronics workers: findings from an electronics enterprise in Taiwan
Source: BMC Public Health. 2021 Aug 9;21:1525. doi: 10.1186/s12889-021-11572-4 (PMC8351339; doi:10.1186/s12889-021-11572-4)
Supplement: Supplementary file 1 — Additional file 1: Questionnaire about shoulder symptom. This is the questionnaire about shoulder symptom developed in this study, including basic information, information on shoulder symptom, and work-related information. Supplementary Table 1: Basic characteristics of study population with and without subacromial impingement syndrome. Supplementary Table 2: Distribution of biomechanical risks for study population with and without subacromial impingement syndrome. Supplementary Table 3: Basic characteristics of study population and distribution of occupational shoulder symptoms. Supplementary Table 4: Distribution of biomechanical risks for occupational shoulder symptoms. Supplementary Table 5: Univariate and multivariate logistic regression analysis of factors influencing occupational shoulder symptoms. [file 12889_2021_11572_MOESM1_ESM.zip › Supplementary Tables 20210710R4.docx]

**Supplementary Table 1** Basic characteristics of study population with and without subacromial impingement syndrome

|  | Subacromial impingement syndrome | | No subacromial impingement syndrome | |  |
| --- | --- | --- | --- | --- | --- |
| Variables | n = 19 | | n = 81 | | p-value |
| Age (years) |  |  |  |  | 0.10 |
| ≦40 | 14 | (73.7%) | 43 | (53.1%) |  |
| >40 | 5 | (26.3%) | 38 | (46.9%) |  |
| Sex^a^ |  |  |  |  | 0.11 |
| Female | 7 | (36.8%) | 16 | (19.8%) |  |
| Male | 12 | (63.2%) | 65 | (80.3%) |  |
| Body height (cm) | 166.2 | (6.7) | 169.3 | (8.1) | 0.42 |
| Body weight (Kg) | 68.8 | (11.8) | 72.4 | (16.6) | 0.59 |
| Body mass index (kg/m^2^) | 24.8 | (3.2) | 25.1 | (4.3) | 0.73 |
| Experience at the job (years) | 5.3 | (6.4) | 6.2 | (7.4) | 0.77 |
| Psychological stress |  |  |  |  | 0.02 |
| Never | 3 | (15.8%) | 11 | (13.6%) |  |
| Some periods | 8 | (42.1%) | 27 | (33.3%) |  |
| Several periods | 6 | (31.6%) | 43 | (53.1%) |  |
| Permanent | 2 | (10.5%) | 0 | (0%) |  |
| Work-related physical fatigue^a^ |  |  |  |  | 0.52 |
| Never or almost never | 1 | (5.3%) | 11 | (13.6%) |  |
| Seldom | 6 | (31.6%) | 22 | (27.2%) |  |
| Quite often | 9 | (47.4%) | 42 | (51.9%) |  |
| Yes, nearly always | 3 | (15.8%) | 6 | (7.4%) |  |

Data are presented as number (%), mean (SD). ^a^The sum of percentage were not 100% due to round off to the first decimal place.

**Supplementary Table 2** Distribution of biomechanical risks for study population with and without subacromial impingement syndrome

|  | Subacromial impingement  syndrome | | No subacromial impingement  syndrome | |  |
| --- | --- | --- | --- | --- | --- |
| Variables | n = 19 | | n = 81 | | p-value |
| **Repetition risk** |  |  |  |  |  |
| Repeating the same motions every few seconds | 9 | (47.4%) | 49 | (60.5%) | 0.30 |
| A sequence of movements repeated more than twice per minute | 9 | (47.4%) | 49 | (60.5%) | 0.30 |
| More than 50% of the cycle time involved in performing the same sequence of motions | 11 | (57.9%) | 54 | (66.7%) | 0.47 |
|  |  |  |  |  |  |
| **Posture risk** |  |  |  |  |  |
| Large range of joint movement such as side to side or up and down | 4 | (21.0%) | 17 | (21.0%) | 0.99 |
| Awkward or extreme joint positions | 4 | (21.1%) | 21 | (25.9%) | 0.66 |
| Joints held in fixed positions | 9 | (47.4%) | 55 | (67.9%) | 0.09 |
| Stretching to reach items or controls | 8 | (42.1%) | 33 | (40.7%) | 0.91 |
| Twisting or rotating items or controls | 7 | (36.8%) | 60 | (74.1%) | <0.01 |
| Working overhead | 5 | (26.3%) | 27 | (33.3%) | 0.56 |
|  |  |  |  |  |  |
| **Force risk** |  |  |  |  |  |
| Pushing, pulling, moving things (including with the fingers or thumb) | 9 | (47.4%) | 48 | (59.3%) | 0.35 |
| Grasping/gripping | 11 | (57.9%) | 48 | (59.3%) | 0.91 |
| Pinch grips i.e. holding or grasping objects between thumb and finger | 9 | (47.4%) | 38 | (46.9%) | 0.97 |
| Steadying or supporting items or work pieces | 5 | (26.3%) | 38 | (46.9%) | 0.10 |
| Shock and/or impact being transmitted to the body from tools or equipment | 3 | (15.8%) | 32 | (36.9%) | 0.05 |
| Objects creating localized pressure on any part of the upper limb | 6 | (31.6%) | 32 | (39.5%) | 0.52 |
|  |  |  |  |  |  |
| **Vibration risk** |  |  |  |  |  |
| Use any powered hand-held or hand-guided tools or equipment/ hand-feed work pieces to vibrating equipment | 3 | (15.8%) | 17 | (21.0%) | 0.61 |

**Supplementary Table 3** Basic characteristics of study population and distribution of occupational shoulder symptoms

|  | Shoulder symptoms | | No shoulder symptoms | |  |
| --- | --- | --- | --- | --- | --- |
| Variables | n = 45 | | n = 686 | | p-value |
| Age (years) |  |  |  |  | 0.32 |
| ≦40 | 28 | (62.2%) | 475 | (69.2%) |  |
| >40 | 17 | (37.8%) | 211 | (30.8%) |  |
| Sex |  |  |  |  |  |
| Female | 22 | (31.9%) | 219 | (28.2%) | 0.51 |
| Male | 47 | (68.1%) | 558 | (71.8%) |  |
| Body height (cm) | 167.0 | (9.3) | 167.2 | (8.5) | 0.77 |
| Body weight (Kg) | 69.1 | (13.1) | 69.6 | (13.8) | 0.70 |
| Body mass index (kg/m^2^) | 24.6 | (3.2) | 24.8 | (4.1) | 0.74 |
| Experience at the job (years) | 5.9 | (5.2) | 5.2 | (5.3) | 0.20 |
| Psychological stress^a^ |  |  |  |  | 0.03 |
| Never | 3 | (6.7%) | 78 | (11.4%) |  |
| Some periods | 13 | (28.9%) | 293 | (42.7%) |  |
| Several periods | 22 | (48.9%) | 271 | (39.5%) |  |
| Permanent | 7 | (15.6%) | 44 | (6.4%) |  |
| Work-related physical fatigue^a^ |  |  |  |  | <0.01 |
| Never or almost never | 1 | (2.2%) | 55 | (8.0%) |  |
| Seldom | 13 | (28.9%) | 289 | (42.1) |  |
| Quite often | 23 | (51.1%) | 298 | (43.4%) |  |
| Yes, nearly always | 8 | (17.8%) | 44 | (6.4%) |  |

Data are presented as number (%), mean (SD). ^a^The sum of percentage were not 100% due to round off to the first decimal place.

**Supplementary Table 4** Distribution of biomechanical risks for occupational shoulder symptoms

|  | Shoulder symptoms | | No shoulder symptoms | |  |
| --- | --- | --- | --- | --- | --- |
| Variables | n = 45 | | n = 686 | | p-value |
| **Repetition risk** |  |  |  |  | <0.01 |
| Repeating the same motions every few seconds | 30 | (66.7%) | 221 | (32.2%) | <0.01 |
| A sequence of movements repeated more than twice per minute | 31 | (68.9%) | 242 | (35.3%) | <0.01 |
| More than 50% of the cycle time involved in performing the same sequence of motions | 34 | (75.6%) | 269 | (39.2%) |  |
|  |  |  |  |  |  |
| **Posture risk** |  |  |  |  |  |
| Large range of joint movement such as side to side or up and down | 14 | (31.1%) | 115 | (16.8%) | 0.01 |
| Awkward or extreme joint positions | 14 | (31.1%) | 65 | (9.5%) | <0.01 |
| Joints held in fixed positions | 27 | (60.0%) | 187 | (27.3%) | <0.01 |
| Stretching to reach items or controls | 24 | (53.3%) | 225 | (32.8%) | <0.01 |
| Twisting or rotating items or controls | 22 | (48.9%) | 201 | (29.3%) | <0.01 |
| Working overhead | 14 | (31.1%) | 92 | (13.4%) | <0.01 |
|  |  |  |  |  |  |
| **Force risk** |  |  |  |  |  |
| Pushing, pulling, moving things (including with the fingers or thumb) | 27 | (60.0%) | 272 | (39.7%) | <0.01 |
| Grasping/gripping | 24 | (53.3%) | 265 | (38.6%) | 0.05 |
| Pinch grips i.e. holding or grasping objects between thumb and finger | 17 | (37.8%) | 186 | (27.1%) | 0.12 |
| Steadying or supporting items or work pieces | 17 | (37.8%) | 157 | (22.9%) | 0.02 |
| Shock and/or impact being transmitted to the body from tools or equipment | 6 | (13.3%) | 55 | (8.0%) | 0.21 |
| Objects creating localized pressure on any part of the upper limb | 11 | (24.4%) | 73 | (10.6%) | <0.01 |
|  |  |  |  |  |  |
| **Vibration risk** |  |  |  |  |  |
| Use any powered hand-held or hand-guided tools or equipment/ hand-feed work pieces to vibrating equipment | 7 | (15.6%) | 61 | (8.9%) | 0.14 |

**Supplementary Table 5** Univariate and multivariate logistic regression analysis of factors influencing occupational shoulder symptoms

|  | Shoulder symptoms | | No shoulder symptoms | | Univariate analysis | | | Multivariate model^d^ | | |
| --- | --- | --- | --- | --- | --- | --- | --- | --- | --- | --- |
|  | n | % | n | % | OR | 95%CI | p-value | OR | 95%CI | p-value |
| Age (years) |  |  |  |  |  |  | 0.33 |  |  |  |
| > 40^e^ |  |  |  |  | 1 | - |  |  |  |  |
| ≦40 |  |  |  |  | 1.37 | 0.73-2.55 |  |  |  |  |
| Sex |  |  |  |  |  |  | 0.34 |  |  |  |
| Male^e^ |  |  |  |  | 1 | - |  |  |  |  |
| Female |  |  |  |  | 1.36 | 0.72-2.56 |  |  |  |  |
| Body mass index (kg/m^2^) |  |  |  |  | 0.99 | 0.92-1.07 | 0.80 |  |  |  |
| Psychological stress |  |  |  |  |  |  | 0.04 |  |  | 0.67 |
| Never^e^ |  |  |  |  | 1 | - |  | 1 | - |  |
| Some periods |  |  |  |  | 1.15 | 0.32-4.15 |  | 0.05 | 0.10-2.42 |  |
| Several periods |  |  |  |  | 2.11 | 0.62-7.24 |  | 0.81 | 0.16-4.07 |  |
| Permanent |  |  |  |  | 4.14 | 1.02-16.81^a^ |  | 0.73 | 0.10-5.31 |  |
| Work-related physical fatigue |  |  |  |  |  |  | 0.01 |  |  | 0.45 |
| Never or almost never^e^ |  |  |  |  | 1 | - |  | 1 | - |  |
| Seldom |  |  |  |  | 2.47 | 0.32-19.30 |  | 4.63 | 0.39-55.47 |  |
| Quite often |  |  |  |  | 4.25 | 0.56-32.09 |  | 5.21 | 0.41-66.29 |  |
| Yes, nearly always |  |  |  |  | 10.00 | 1.21-83.00^a^ |  | 9.39 | 0.59-149.53 |  |
| Repetition risk |  |  |  |  |  |  | <0.01 |  |  | 0.02 |
| No^e^ | 10 | 22.2 | 376 | 54.8 | 1 | - |  | 1 | - |  |
| Low | 7 | 15.6 | 131 | 19.1 | 2.01 | 0.75-5.39 |  | 1.22 | 0.39-3.79 |  |
| High | 28 | 62.2 | 179 | 26.1 | 5.88 | 2.80-12.37^c^ |  | 3.09 | 1.18-8.08^a^ |  |
| Posture risk |  |  |  |  |  |  | <0.01 |  |  | 0.02 |
| No^e^ | 8 | 17.8 | 359 | 52.3 | 1 | - |  | 1 | - |  |
| Low | 14 | 31.1 | 175 | 25.5 | 3.59 | 1.48-8.72^b^ |  | 3.18 | 1.06-9.51^a^ |  |
| High | 23 | 51.1 | 152 | 22.2 | 6.79 | 2.97-15.52^c^ |  | 6.00 | 1.76-20.41^b^ |  |
| Force risk |  |  |  |  |  |  | 0.03 |  |  | 0.17 |
| No^e^ | 16 | 35.6 | 358 | 52.2 | 1 | - |  | 1 | - |  |
| Low | 8 | 17.8 | 137 | 20.0 | 1.31 | 0.55-3.12 |  | 0.46 | 0.17-1.24 |  |
| High | 21 | 46.7 | 191 | 27.8 | 2.46 | 1.25-4.83^b^ |  | 0.44 | 0.17-1.11 |  |
| Vibration risk |  |  |  |  |  |  | 0.14 |  |  |  |
| No^e^ | 38 | 84.4 | 625 | 91.1 | 1 | - |  |  |  |  |
| Yes | 7 | 15.6 | 61 | 8.9 | 1.89 | 0.81-4.41 |  |  |  |  |

OR, odds ratio; CI, conﬁdence interval; ^a^p<0.05, ^b^p<0.01, ^c^p<0.0001; ^d^Multivariable model included: independence of risk factors in the univariate analysis and no model selection; ; ^e^Reference group.
